# Comparison of different T cell assays for the retrospective determination of SARS-CoV-2 infection

**DOI:** 10.1099/jgv.0.002055

**Published:** 2024-12-20

**Authors:** Benjamin Krishna, Marina Metaxaki, Marianne Perera, Mark Wills, Nyarie Sithole

**Affiliations:** 1 Cambridge Institute of Therapeutic Immunology and Infectious Disease (CITIID), Cambridge, UK; 2 Division of Virology, Department of Pathology, University of Cambridge, Cambridge, UK

**Keywords:** COVID-19, peptide, SARS-CoV-2, T cell

## Abstract

It is important to be able to retrospectively determine severe acute respiratory syndrome coronavirus 2 (SARS-CoV-2) infections with high accuracy, both for post-coronavirus disease 2019 (COVID-19) epidemiological studies, and to distinguish between Long COVID and other multi-syndromic diseases that have overlapping symptoms. Although serum antibody levels can be measured to retrospectively diagnose SARS-CoV-2 infections, peptide stimulation of memory T cell responses is a more sensitive approach. This is because robust memory T cells are generated after SARS-CoV-2 infection and persist even after antibodies wane below detectability thresholds. In this study, we compare T cell responses using FluoroSpot-based methods and overnight stimulation of whole blood with SARS-CoV-2 peptides followed by an ELISA. Both approaches have comparable sensitivity and specificity but require different equipment and samples to be used. Furthermore, the elimination of peptides that cross-react with other coronaviruses increases the assay specificity but trades off some sensitivity. Finally, this approach can be used on archival, cryopreserved PBMCs. This work shows comparative advantages for several methods to measure SARS-CoV-2 T cell responses that could be utilized by any laboratory studying the effects of the coronavirus disease 2019 pandemic.

## Data availability

Anonymized data are available upon request. Please contact the corresponding author, Nyarie Sithole.

## Introduction

Severe acute respiratory syndrome coronavirus 2 (SARS-CoV-2) infections continue to be a major source of death and disability worldwide. Although vaccinations have reduced the severity of coronavirus disease 2019 (COVID-19) pathology [1,5], older and immunocompromised people may still suffer from severe disease, resulting in prolonged hospitalizations and/or death [6]. Waning immunity post-vaccination combined with the emergence of significant new variants means that boosters and new vaccine designs will be needed for the foreseeable future to protect the population from more severe disease [7,8].

On top of acute illness, COVID-19 leaves a significant proportion of patients with a plethora of conditions termed ‘post-acute sequelae of COVID-19’, post-COVID syndrome or ‘Long COVID’ [9,14]. Long COVID is associated with a myriad of symptoms and sequelae that occur after acute COVID-19 [15].

A key prerequisite for the diagnosis of Long COVID is evidence of SARS-CoV-2 infection, providing objective evidence to distinguish between Long COVID and what could be a result of other post-infectious syndromic conditions [16]. Although both lateral flow and reverse transcription quantitative polymerase chain reaction (RT-qPCR) testing are available, both are underused, experience false negatives, are dependent on the user for good sampling technique and are not always available and the results may not be recorded [17,18]. Determining past SARS-CoV-2 exposure is also useful for future epidemiology efforts, so researchers can delineate SARS-CoV-2 infection status as an important correlate of other health outcomes such as cardiovascular disease [11,19], infections with other viruses and reinfection with SARS-CoV-2. If applied retrospectively, this would also allow us to analyse samples taken before/during the pandemic for SARS-CoV-2 exposure. Finally, another viral pandemic is almost inevitable [20], which will be followed by post-viral syndromes similar to Long COVID. Understanding now how best to retrospectively diagnose viral infections may give researchers a head start before the next pandemic occurs.

Although serum antibody levels can be used to determine historic SARS-CoV-2 infection, spike antibodies can no longer be used in vaccinated individuals as they will also have memory immune responses to spike. Antibody responses to nucleocapsid (Nc) could be used as this is not a component of most vaccines; however, these antibodies also tend to wane over time, increasing the risk of false-negative results [21,27]. T cell-based assays, such as FluoroSpot and Enzyme-linked immunosorbent spot (ELISpot) assays, show higher sensitivity to past SARS-CoV-2 infection compared to antibody serology assays [28,31].

To quantify T cell responses, we stimulated whole blood samples with SARS-CoV-2 peptides and then measured IL-2 and IFN-γ by ELISA. The entire assay can be performed with 3.5 ml of blood, which is advantageous for patients who are difficult to bleed. Whole blood peptide stimulation has been used previously to analyse T cell responses to SARS-CoV-2 but has focussed on IFN-γ production in response to spike peptides [32], or spike, Nc and membrane (M) peptides all together [33,37] or Nc peptide alone [38]. Others have used different readouts such as flow cytometry to measure IFN-γ production [39] or quantitative PCR of CXCL10 after whole blood stimulation with peptides from spike protein [40].

Previous work using FluoroSpot assays showed that analysing both Nc and M responses separately improved the specificity of the assay [30]. Applying this approach to whole blood peptide stimulation showed 88.9% sensitivity and 85.1% specificity, similar to the results using FluoroSpot assays. Interestingly, IFN-γ production was a better readout when using the whole blood ELISA approach compared to IL-2 when performing FluoroSpot assays. This highlights the necessity to optimize readout cytokines for different methodologies.

Previous FluoroSpot analyses of SARS-CoV-2 memory immune responses experienced false positives due to cross-reactivity with seasonal coronaviruses, an issue that has been reported in whole blood peptide assays as well [30,34,36, 38, 41]. To improve upon previous approaches, the Nc and M peptide pools were redesigned to eliminate peptides that cross-react with seasonal coronaviruses. These peptides improved the specificity while trading off some sensitivity, which may open the possibility of having equivocal and unequivocal results.

Furthermore, we have repurposed the whole blood peptide stimulation assay for use with frozen PBMC samples. While FluoroSpot assays can be applied to frozen PBMC samples taken before/during the pandemic, whole blood peptide stimulation requires fresh blood samples. Given that many laboratories across the world have PBMC samples frozen before and during the pandemic, we show here that this same peptide stimulation approach can be applied to historic PBMC samples, allowing broader application of the assay.

## Methods

### Recruitment criteria

**Fig. 1.**
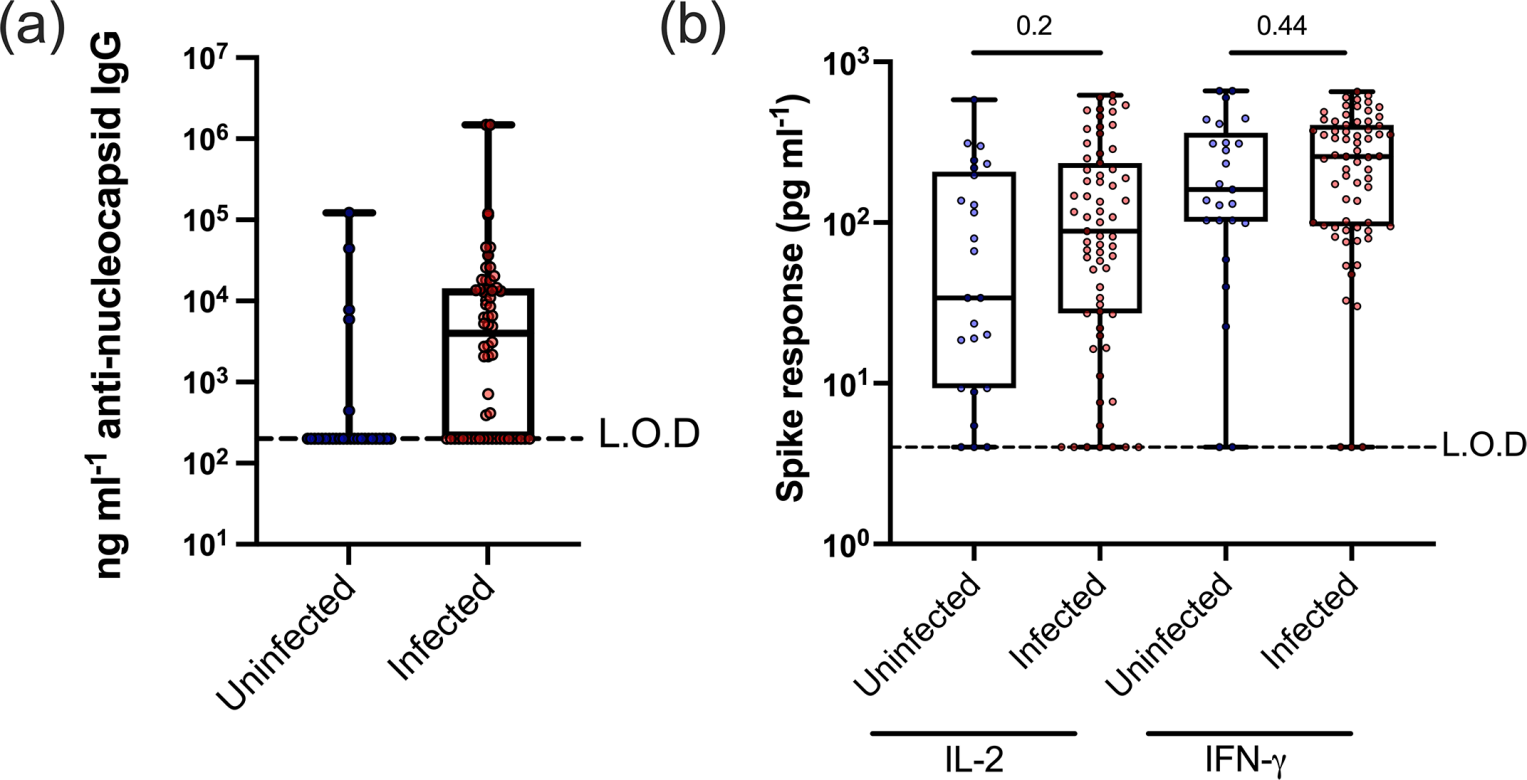
Confirmation of SARS-CoV-2 seronegative status and ability to respond to peptide stimulation. (**a**) To confirm that the uninfected control donors (those with no evidence of infection, *n*=29) had not been exposed to SARS-CoV-2 infection, anti-Nc antibody was measured by ELISA from diluted plasma, and the four donors with anti-NC levels above the median for the infected controls (*n*=52) were excluded from further analysis. Samples that were below the detectability threshold were set to 200 ng µl^−1^. (**b**) A total of 0.5 ml of fresh venous blood was treated with peptide pools covering the entirety of SARS-CoV-2 spike protein (Wuhan strain) at a final concentration of 1 µg ml^−1^/peptide or untreated control wells. Blood was incubated overnight at 37 °C and plasma was harvested the next day. IL-2 and IFN-γ production was measured by ELISA analysis and was plotted here as the difference between spike peptide pool stimulated and untreated negative control. Values at or below 0 were plotted as 0.1 to allow their visualization on logarithmic axes. *P*-values between those with and without evidence of infection were calculated using Mann–Whitney U tests.

All donors gave written informed consent in accordance with the Declaration of Helsinki. All 29 uninfected control donors were bled between November 2021 and February 2022, recruited by the NIHR BioResource Centre Cambridge through the ARIA study with ethical approval from the Cambridge Human Biology Research Ethics Committee (HBREC.2014.07). Participants were excluded from the study if they were being treated with oral or intravenous immunomodulatory drugs (including steroids, tacrolimus, cyclosporins, azathioprines, mycophenolate, methotrexate, rituximab and cyclophosphamide) within the last 3 months, undergoing injected anti-TNF treatments for rheumatoid arthritis and anyone receiving current or recent (last 24 months) cancer chemotherapy. At the time of blood donation, these patients had never had a positive RT-qPCR or lateral flow test result for SARS-CoV-2 and did not report having had symptoms consistent with COVID-19, e.g. anosmia. Of the 25 participants, 8 were part of an asymptomatic screening programme at the University of Cambridge and received regular RT-qPCR tests for SARS-CoV-2 infections, never receiving a positive test result. All participants had received at least two doses of SARS-CoV-2 vaccine at the time of bleeding. Participants were also tested for anti-nucleocapsid (anti-Nc) antibody, and four donors with anti-Nc levels higher than the median for infected donors were excluded from further analysis (Fig. 1). Of the 25 uninfected donors, 8 donors were later infected between March and June 2022 in the UK, most likely with the Omicron variant of SARS-CoV-2.

Sixty-seven infected controls were recruited where ethical approval was obtained from the East of England – Cambridge Central Research Ethics Committee (‘NIHR BioResource’ REC ref 17/EE/0025) as described by Bergamaschi *et al*. [42] or under the Cambridge COVID-19 NIHR BioResource joint Consent Form [Research Ethics Committee (NRES number (REC)) no. T1gC1] study NBR87. These patients had received a positive RT-qPCR result for SARS-CoV-2 and donated blood 1–28 months after positive test results (median=7, interquartile range 6–18 months). All donors had received at least two doses of SARS-CoV-2 vaccine at the time of bleeding. Infections were between March 2020 and January 2022 in the UK and so would have been from Wuhan, Alpha and Delta variants of SARS-CoV-2. Of the infected cohort, one donor was in hospital with COVID-19, while the rest were symptomatic but did not require hospital treatment. Within this cohort, none reported a second RT-qPCR-confirmed SARS-CoV-2 infection before bleeding. Four donors were later excluded from the study as their T cells did not respond to the phytohaemagglutinin (PHA)-positive control treatment.

### Measurement of anti-Nc antibody levels

Anti-Nc antibody was measured using LEGEND MAX SARS-CoV-2 Nucleocapsid kit following manufacturer’s instructions. In brief, plasma from each donor was diluted 1/2000 to bring samples to within the assay’s standard range and then applied to the pre-coated plate. Anti-Nc antibodies were detected using biotinylated detection antibody and avidin-HRP (ELISA). Absolute levels of anti-Nc antibody were quantified against the standard curve.

Peptide pools covering the entirety of the spike, M and Nc proteins from SARS-CoV-2 (Wuhan variant) have been previously described [30,43]: ‘A peptide pool was generated using the following: 1. PepTivator SARS-CoV-2 Prot_S containing the sequence domains aa 304–338, 421–475, 492–519, 683–707, 741–770, 785–802, and 885–1273 and S1 N-terminal S1 domain of the surface glycoprotein (“S”) of SARS-Coronavirus 2 (GenBank MN908947.3, Protein QHD43416.1). 2. The PepTivator SARS-CoV-2 Prot_S1 containing the aa sequence 1–692. The peptides used are 15aa amino acids with 11 amino acid overlaps.’ In addition to these, we also used PepTivator SARS-CoV-2 Prot_N covering the entire sequence of Nc (GenBank MN908947.3, Protein QHD43423.2) and PepTivator SARS-CoV-2 Prot_M covering the entire sequence of M (GenBank MN908947.3, Protein QHD43419.1). In addition to these, ‘epitope-restricted peptide pools’ were generated, which excluded peptides with more than six sequential aa overlaps with circulating human coronaviruses: NL63, 229E, OC43 or HKU1. These pools were ordered bespoke as a PEPscreen peptide library from ProImmune. Lyophilized powder was resuspended in 80% DMSO with 20% TexMACS media to give a concentration of 40 mg ml^−1^ and incubated at 37 °C for 1 h. Individual peptides were then pooled and diluted in TexMACS media to 50 µg ml^−1^/peptide stock solution, which was then used at 1 µg ml^−1^/peptide final concentration.

Venous blood (3.5 ml) was collected from patients into heparin tubes. 0.5 ml aliquots of blood were stimulated with peptide pools (1 µg ml^−1^/peptide), covering the entirety of spike, M or Nc proteins from SARS-CoV-2, mock treated with PBS containing 0.1% DMSO as a negative control or treated with PHA (1 µg ml^−1^) as a positive control. Where relevant, samples were also treated with the epitope-restricted peptide pools of Nc or M proteins. Samples were incubated overnight at 37 °C with 5% CO_2_ and then centrifuged at 500 ***g*** for 5 min to precipitate blood cells. Plasma was collected and frozen at −80 °C.

PBMC samples, frozen at 10^7^ cells per vial, were thawed (one vial per donor) in TexMACS media containing 10 U ml^−1^ DNAse I (Roche, 4716728001) for 1 h at 37 °C with 5% CO_2_. The cells were then washed twice in PBS and plated in TexMACS at 2.5×10^5^ cells per well, with 100 pg ml^−1^ anti-CD28 antibody (Mabtech 3608-1-50). Peptide stimulation was overnight at 37 °C at 1 µg ml^−1^/peptide, while negative controls were media treated and positive controls were treated with PHA (1 µg ml^−1^) and anti-CD3 antibody (1 µg ml^−1^, Mabtech 3605-1-1000). Post-incubation, plates were centrifuged at 1200 ***g*** for 10 min, and the supernatant was collected and then frozen at −80 °C until analysis by ELISA.

ELISAs for IL-2 and IFN-γ were performed using ELISA MAX Deluxe Sets from BioLegend (Cat numbers 431801 and 430101, respectively), following the manufacturer’s protocols. In short, plates were coated overnight in antibodies at 4 °C. On the next day, they were washed 3× in PBS/Tween 20 and then blocked in PBS with 1% BSA for 1 h at room temperature. Plates were washed again 3× in PBS/Tween 20, and then, samples and standards were added after 1 : 4 dilution in PBS with 1% BSA (or 1 : 2 dilution for media from PBMC cultures). After 2-h incubation at room temperature, the plates were washed again and the HRP-conjugated detection antibody was added and incubated for 30 min at room temperature. Plates were then developed using 3,3′,5,5′-Tetramethylbenzidine reagent (77247, 77248, BioLegend) for 15 min at room temperature, and the reaction was stopped with 2M sulphuric acid.

### FluoroSpot

To allow direct comparison between publications, FluoroSpot assays were performed identically to our previously published work [30], using TexMACS media instead of RPMI, as recommended to reduce background T cell activation [44]:

“PBMCs were isolated from citrated blood samples by layering blood onto Lymphoprep (Axis-Shield, Oslo, Norway) and performing density gradient centrifugation at 1200 ***g*** for 10 min. PBMCs at the interface were collected and washed 2× in PBS.

TexMACS was used in this study as has been previously used by our group and others [43,45]. 2×10^5^ PBMCs suspended in TexMACS (Miltenyi Biotec) supplemented with 5% human AB serum (Sigma-Aldrich) were incubated on FluoroSpot plates coated with human IFN-γ and IL-2 antibodies [FluoroSpot (Mabtech AB, Nacka Strand, Sweden)] in duplicate with ORF mix peptides (final peptide concentration of 1 µg ml^−1^/peptide) as well as a TexMACS-only negative control and positive control mix [containing anti-CD3 (Mabtech AB; RRID:AB_907218), *Staphylococcus* enterotoxin B and lipopolysaccharide (all Sigma-Aldrich)] at 37 °C in a humidified CO_2_ atmosphere for 48 h. The cells and medium were decanted from the plate, and the assay was developed following the manufacturer’s instructions. Developed plates were read using an AID iSpot reader (Oxford Biosystems, Oxford, UK) and counted using AID EliSpot v7 software (Autoimmun Diagnostika GmbH, Strasberg, Germany) using distinct counting protocols for IFN-γ and IL-2 secretion. Donor results were discounted from further analysis if there was less than 100 spot forming units (SFUs) in the positive control relative to the background SFU. All data were then corrected for background cytokine production.”

Data were determined to be non-parametric using the Shapiro–Wilk test. Significance was then tested using Mann–Whitney U or Wilcoxon ranked sign test as indicated in the manuscript using Prism v9.5.1.

## Results

### Utility of SARS-CoV-2 T cell responses in COVID-19 antibody-negative donors

Peptide stimulation of whole blood leads to the production of cytokines by antigen-specific T cells [36]. We aimed to test whether, after separating out the spike, M and Nc peptide pools into separate tubes, cytokine responses to peptide stimulation were sufficiently robust and consistent to allow determination of past SARS-CoV-2 infection. To start, fresh whole blood samples were collected from volunteers who had RT-qPCR-confirmed SARS-CoV-2 infections at least 1 month prior (infected cohort) and from a group of volunteers who suspected that they had not previously been infected with SARS-CoV-2 (uninfected cohort). This cohort never had a positive COVID-19 result from either a lateral flow test or RT-qPCR test and had no reported symptoms consistent with COVID-19 before blood was taken. These samples were taken between November 2021 and February 2022.

To help confirm that the uninfected cohort had not previously been infected with SARS-CoV-2, all donors were tested for anti-Nc antibodies (Fig. 1a). We excluded four donors who had anti-Nc antibody levels, which were higher than the median for the infected controls, as they were likely to have been asymptomatically infected without being tested. Sixty-seven per cent of tested infected donors (39/58) had anti-Nc IgG levels above the limit of detectability.

Subsequently, to confirm that peptide stimulation using a small volume of blood, 0.5 ml, was sufficient to produce detectable IL-2 and IFN-γ production using standard ELISAs, blood samples were stimulated with spike peptide pools covering the entirety of the Wuhan spike protein. As these donors had been vaccinated twice against SARS-CoV-2, they all would be expected to produce a detectable response and therefore act as a positive control.

We found that 48/52 volunteers produced IFN-γ and 44/52 produced detectable IL-2 and 41/52 had both IL-2 and IFN-γ responses. This experiment also tested whether the magnitude of IL-2 or IFN-γ production was higher in those who had previously been vaccinated and infected with SARS-CoV-2 compared to those who had been vaccinated and not infected. There was no significant difference in the magnitude of IL-2 or IFN-γ production between the infected and uninfected cohorts (Fig. 1b). Three donors responded with neither IL-2 nor IFN-γ to spike peptide stimulation, and these were excluded based on this criterion from further analysis.

### IL-2 and IFN-γ production after whole blood stimulation with M and Nc peptides discriminates between known infected and suspected uninfected donors

**Fig. 2.**
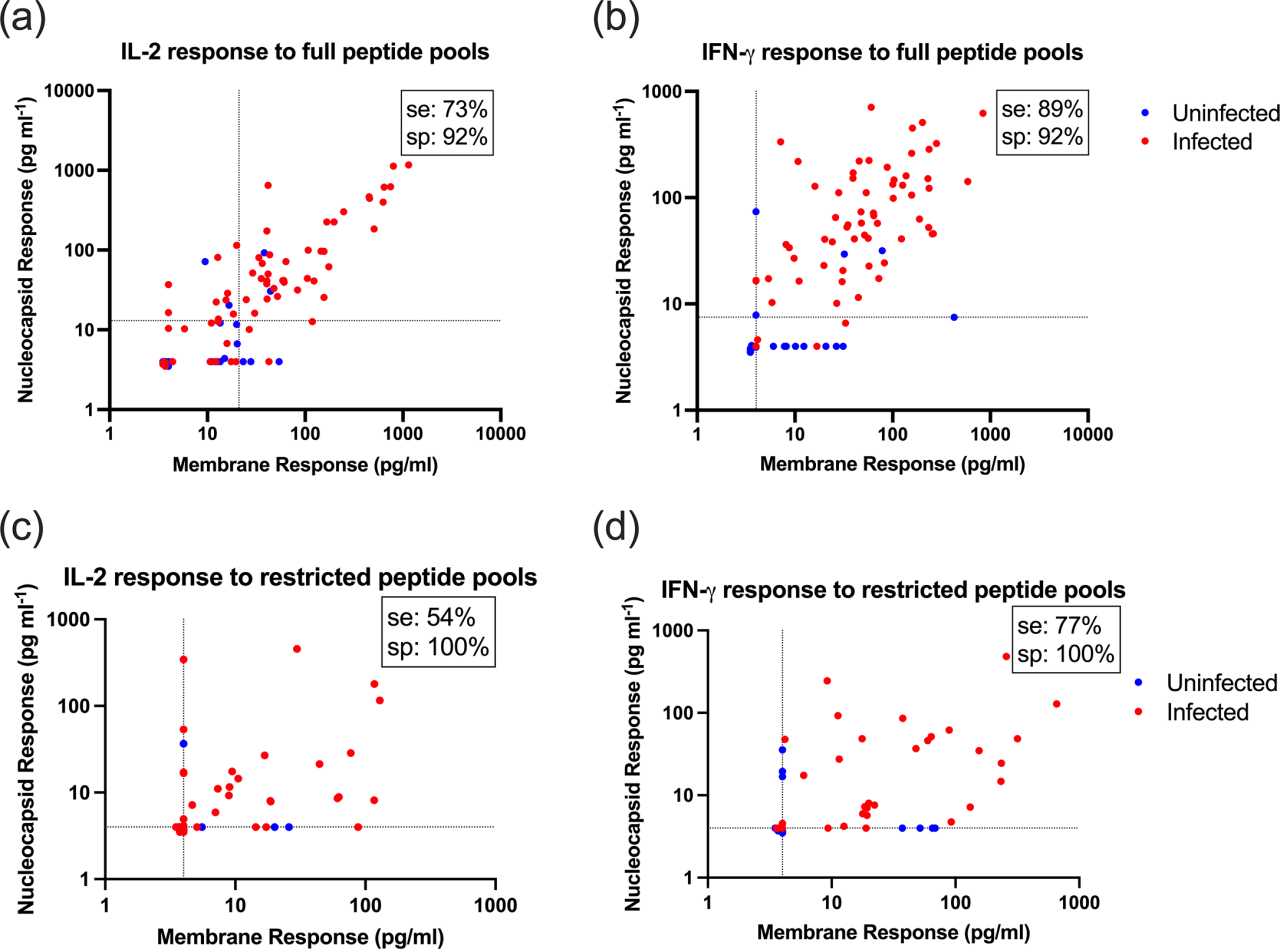
IL-2 and IFN-γ production after whole blood peptide stimulation discriminates between known infected and suspected uninfected donors, while removing cross-reactive epitopes improves the specificity (sp). A total of 0.5 ml of fresh venous blood from uninfected (*n*=25) and infected (*n*=52) donors was treated with peptide pools covering M or Nc SARS-CoV-2 proteins at a final concentration of 1 µg ml^−1^/peptide or untreated control. Blood was incubated overnight at 37 °C and plasma was harvested the next day by centrifugation. IL-2 and IFN-γ production to Nc and M peptides was measured by ELISA analysis and is plotted here as the difference between peptide stimulated and untreated negative controls. Values below the limit of detectability (4 pg µl^−1^) were plotted between 3.5 and 4 to allow their visualization on logarithmic axes without data points superimposing. (a, b) Responses to peptide pools covering the whole Wuhan M and Nc proteins. (c, d) Responses to peptide pools, which exclude epitopes that cross-react with circulating coronaviruses. Dotted lines indicate the thresholds for negative and positive responses based on receiver operating characteristic (ROC) analysis. Numbers in the top right hand corner of each scatter graph indicate the sensitivity (se) and sp of each assay.

Measuring T cell responses to Nc and M peptide pools separately can distinguish infected from uninfected donors with high accuracy [30]. Peptide pools, which cover the entirety of the Nc and M proteins derived from the original Wuhan SARS-CoV-2 variant, were added to fresh blood. The infected cohort (*n*=63) had positive SARS-CoV-2 RT-qPCR tests between 1 and 28 months prior to donating blood. Suspected uninfected donors (*n*=25) had no history of COVID-19 symptoms or positive RT-qPCR, lateral flow tests or anti-Nc antibodies (as confirmed in Fig. 1a) prior to taking blood samples. The stimulation with M and Nc peptides showed a clear increase in IL-2 and IFN-γ responses in known infected compared to uninfected donors (Fig. 2a, b). Thresholds were applied using receiver operating characteristic curve analyses (Fig. S1, available in the online version of this article), suggesting that this test had 73% sensitivity and 92% specificity for IL-2 and 89% sensitivity and 92% specificity for IFN-γ.

Despite this, some of the uninfected cohort showed detectable responses to M and Nc peptide pools, which in a few cases would lead to false-positive results. We therefore hypothesized that this may be caused by T cell cross-reactivity, due to peptides present in the whole Nc and M pools derived from the Wuhan SARS-CoV-2 isolate sequence, which were sufficiently similar in sequence to previous circulating human coronaviruses. If correct, removing these cross-reactive peptides would improve the specificity of the ELISA by reducing false-positive results.

To test this hypothesis, epitope-restricted peptide pools were designed, which excluded any peptides with more than six aa overlaps with the circulating human coronaviruses: HKU1, NL63, 229E and OC43 (Table S1). Whole blood was then treated with these epitope-restricted peptides as before (Fig. 2c, d). Our results showed 77% sensitivity and 100% specificity for IFN-γ and 54% sensitivity and 100% specificity for IL-2. Although some of the uninfected cohort responded to either epitope-restricted M or Nc peptide pools alone, none of the cohort had detectable IL-2 or IFN-γ responses to both epitope-restricted peptide pools. As 4/26 responded to both whole peptide pools with IFN-γ and 7/26 responded with IL-2, the hypothesis that the uninfected control samples can respond to cross-reactive epitopes is supported. However, this approach does decrease the sensitivity of the assay, as some infected cohort donors fail (7/35) to respond to both epitope-restricted Nc and M peptide pools.

### IFN-γ production after whole blood peptide stimulation with peptides based on the Wuhan strain sequences still occurs in those infected with Omicron variants

**Fig. 3.**
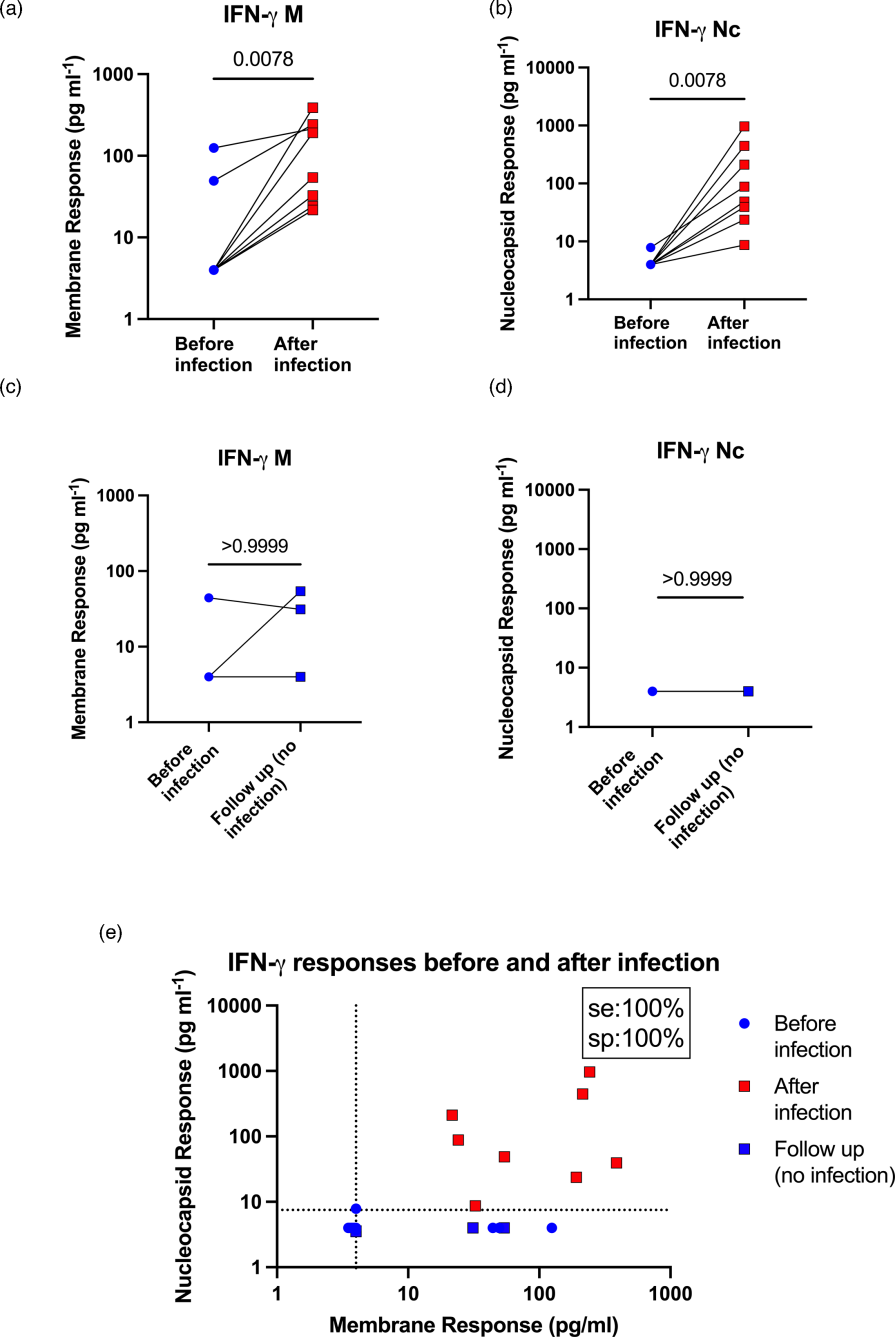
IFN-γ production after whole blood peptide stimulation discriminates before and after infection in the same individuals. Twelve donors were tested using the whole blood peptide assay with full M and Nc peptide pools as in Fig. 2 before and after lateral flow test or RT-qPCR-confirmed SARS-CoV-2 infections. Values at or below 0 were plotted as 4 to allow their visualization on logarithmic axes. (a–d) Results individually for each peptide pool and either with or without infection. Significance is calculated by the Mann–Whitney U test. (e) The same data for M vs Nc responses for the cohort before and after infection.

**Fig. 4.**
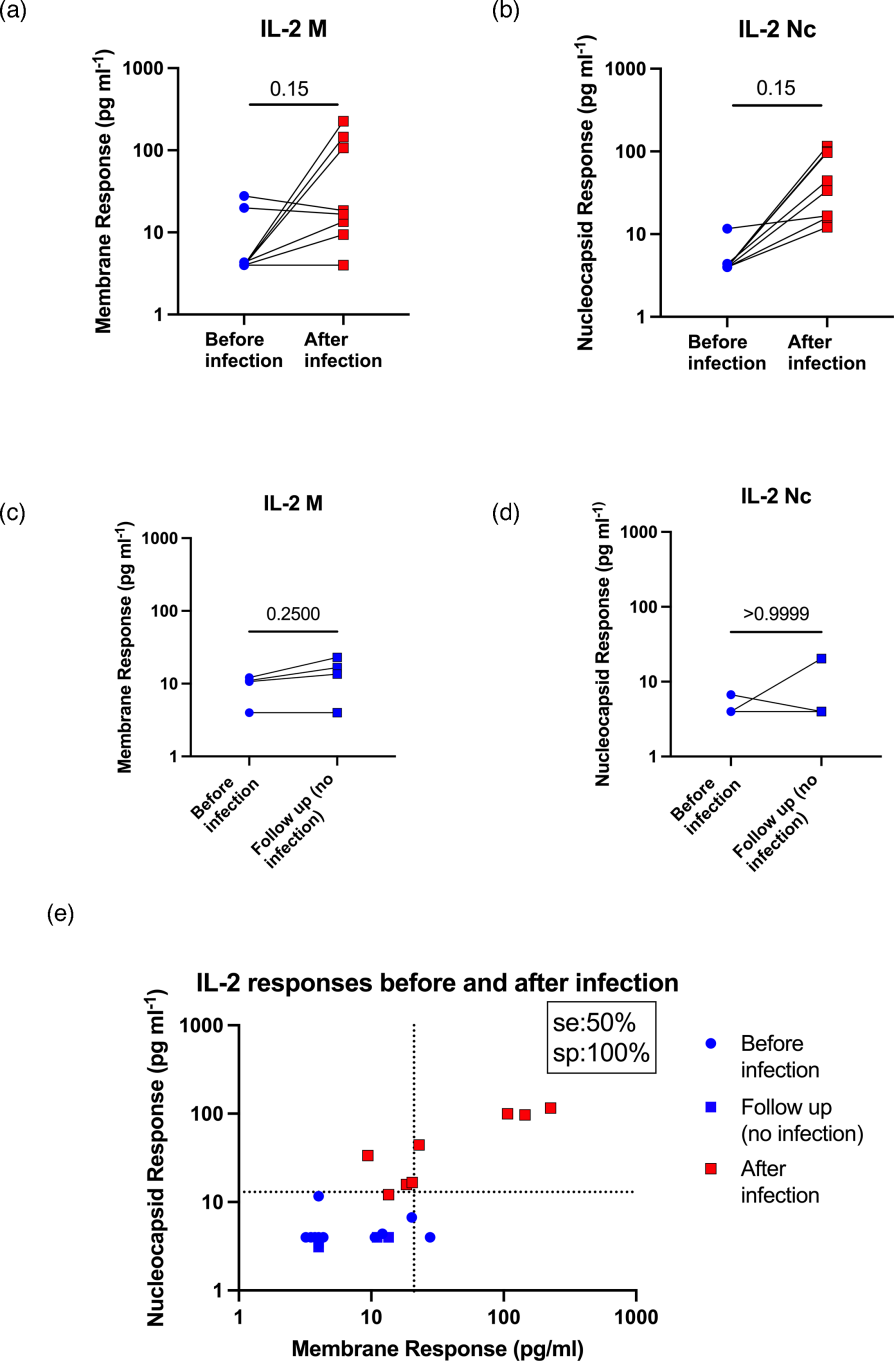
IL-2 production after whole blood peptide stimulation only weakly discriminates before and after infection in the same individuals. Twelve donors were tested using the whole blood peptide assay with full M and Nc peptide pools as in Fig. 2 before and after lateral flow test or RT-qPCR-confirmed SARS-CoV-2 infections. Values at or below 0 were plotted as 4 to allow their visualization on logarithmic axes. (a–d) Results individually for each peptide pool and either with or without infection. Significance is calculated by the Mann–Whitney U test. (e) The same data for M vs Nc responses for the cohort before and after infection.

Between March and June 2022, some uninfected cohort donors that were originally tested (Figs1  2) became infected with SARS-CoV-2, which were very likely Omicron variants as it was the predominant circulating strain at that time. This gave us the opportunity to test the whole blood stimulation assay on the same donors before and after infection, helping to rule out donor-specific variables and confirm that Wuhan peptides could still stimulate memory recall responses in those infected with later variants. Of the 12 donors, 8 were infected and 4 remained uninfected. Infected individuals were bled a second time between 6 and 16 weeks post-infection (median 9.5 weeks). Infection induced a significant increase in IFN-γ responses to M and Nc in infected donors. However, there were unfortunately only four donors as uninfected controls, which is too few for meaningful statistical analysis. Nevertheless, only one uninfected donor showed an increase in their IFN-γ response, which was to M peptide stimulation only (Fig. 3a–d). When responses to both peptides were plotted together (Fig. 3e), the efficacy of this approach can clearly be seen. In agreement with results in Fig. 2, infection with SARS-CoV-2 induced memory IL-2 responses, but these were weaker and less reliable than IFN-γ responses (Fig. 4). We also measured serum anti-Nc IgG levels in all donors twice (Fig. S3). No donor (0/12) had detectable anti-Nc IgG levels before infection, none showed detectable anti-Nc IgG levels if they were not subsequently infected (0/4) and 7/8 donors showed detectable anti-Nc IgG levels after infection.

### Historical cryopreserved PBMC samples can be stimulated with peptides to distinguish previous SARS-CoV-2 infection from vaccination

A disadvantage of using whole blood samples for peptide stimulation is that a fresh blood sample is required for stimulation with peptide pools on the same day that the blood has been taken. PBMCs, which have been cryopreserved and thawed, can be used to determine past SARS-CoV-2 infection by FluoroSpot analysis [30,46, 47]. One advantage of frozen PBMCs is that they can be stored in liquid nitrogen for future use. A second advantage of frozen PBMCs is that many were collected and stored before or during the SARS-CoV-2 pandemic and would therefore be important control samples for future studies on the SARS-CoV-2 pandemic. It is therefore important to test whether cryopreserved PBMCs could be stimulated with SARS-CoV-2 peptide pools to produce sufficient IL-2 and IFN-γ for detection by ELISA and if this method would be comparable to the efficacy of FluoroSpot approaches.

**Fig. 5.**
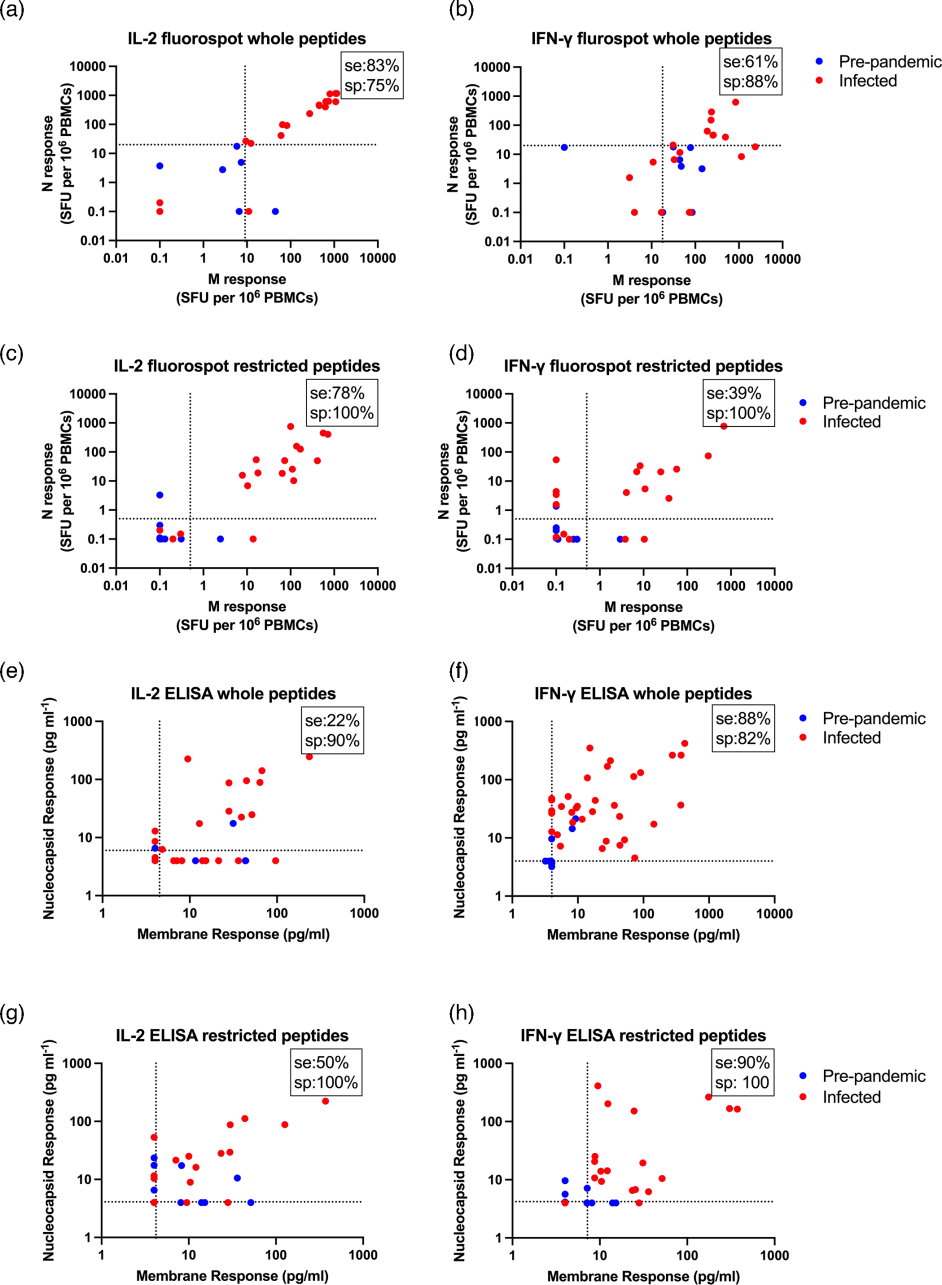
IL-2 and IFN-γ production after stimulation of PBMCs discriminates between known infected and suspected uninfected donors. (**a–d**) To test the efficacy of these epitope-restricted peptide pools by FluoroSpot analysis, PBMCs were isolated from pre-pandemic, unexposed donors (blue, *n*=8) or known infected (RT-qPCR confirmed, red *n*=18) controls. These PBMCs were stimulated with epitope-restricted Nc or M peptide pools (1 µg ml^−1^/peptide) or untreated control. IL-2 and IFN-γ production was measured by FluoroSpot analysis as previously described [51]. Each condition was run in duplicate and the number of SFUs were quantified by a peptide-negative, unstimulated control, which was subtracted to remove background cytokine production. Zero results are set as 0.1 to allow their inclusion on a log scale. (**e–h**) PBMCs from unexposed donors (blue *n*=23) or known infected (RT-qPCR confirmed, red *n*=20) controls were treated with epitope-restricted peptide pools covering M or Nc SARS-CoV-2 proteins at a final concentration of 1 µg ml^−1^/peptide or untreated control. Samples were incubated overnight at 37 °C. Supernatant IL-2 and IFN-γ was measured by ELISA analysis and plotted as in Fig. 2. Dotted lines indicate the thresholds for negative and positive responses based on receiver operating characteristic (ROC) analysis. Numbers in the top right hand corner of each scatter graph indicate the sensitivity (se) and specificity (sp) of each assay.

To confirm that the new, epitope-restricted peptide pool could stimulate PBMCs to produce IL-2 and IFN-γ by FluoroSpot analysis, FluoroSpot assays were used as these are very sensitive and quantitative, allowing comparison of new data with previous work [30]. For this work, PBMCs from RT-qPCR-confirmed infected cohort samples were used as a positive control and compared to PBMCs donated between 2014 and 2019, before the COVID-19 pandemic, and referred to as pre-pandemic samples. Results from this assay were similar to the whole blood assays, showing that some unexposed cohort responded to the whole peptide pools (Fig. 5a, b) but none showed responses to both the SARS-CoV-2 Nc and M epitope-restricted peptide pools (Fig. 5c, d). This confirmed that eliminating the cross-reactive peptides eliminates responses in the uninfected cohort; however, as seen in comparison with Fig. 2, the sensitivity of the assay is reduced.

As there may be situations where ELISAs are advantageous over FluoroSpot-based approaches, it was important to demonstrate that peptide stimulation of cultured PBMCs produced detectable IFN-γ and IL-2 responses by ELISA. To achieve this, thawed PBMCs were cultured with the whole peptide pools (Fig. 5e, f) or epitope-restricted peptide pools (Fig. 5g, h). Peptide stimulation produced robust IFN-γ and IL-2 responses (Fig. 5e–h); however, IFN-γ production (specificity: 93.3%, sensitivity 88.2%) was a better predictor of past SARS-CoV-2 infection than IL-2 (specificity: 58.8%, sensitivity 86.7%) in this system. Similar to the results in fresh whole blood assays and by FluoroSpot analysis, epitope-restricted peptide pools stimulated lower responses in infected controls and no unexposed controls produced IFN-γ responses to both Nc and M peptide pools. Only 2/23 produced IL-2 (Fig. 5g, h).

## Discussion

As SARS-CoV-2 causes a plethora of post-acute COVID-19 conditions, it is necessary to consider both SARS-CoV-2 exposure and T cell memory immune responses to SARS-CoV-2 when studying epidemiology of cardiac diseases, other viral infections, COVID variants, vaccine efficacy and Long COVID. There is, therefore, an unmet need for efficient and reliable methods to measure the presence and strength of *ex vivo* T cell responses to SARS-CoV-2 at least in part to determine SARS-CoV-2 exposure.

Although antibody serology is able to ascertain past SARS-CoV-2 infection with some accuracy, multiple publications have now found that T cell-based assays demonstrate superior sensitivity [28,31,48] and that T cell responses tend to be more durable than antibody levels [49,51], although one study was an exception to this, finding stronger antibody levels than IFN-γ T cell responses to spike peptide pools in five infected healthcare workers prior to vaccination [52]. In line with these other studies, 67% of tested infected donors in this study had anti-Nc IgG levels above the limit of detectability (Fig. 1a), but 89% of these donors had a positive IFN-γ response to Nc and M peptide stimulation (Fig. 2b).

This study first demonstrated that stimulating fresh blood with peptides from SARS-CoV-2 followed by ELISA analysis of IFN-γ or IL-2 (Figs1  2) has similar efficacy to FluoroSpot analyses, confirming observations by many others that this approach works well for measuring T cell responses [32,40]. Given that this approach is also effective for capillary blood derived from a finger prick [35,53], there is promise for future diagnostic assays using minimal blood.

This whole blood stimulation assay produced a bimodal distribution of IL-2 responses to spike peptide stimulation (Fig. 1b). As other groups who used whole blood peptide stimulation did not measure IL-2 production by ELISA, we cannot easily compare this observation to other publications; however, we did not see a bimodal distribution of IL-2 responses to spike peptides using FluoroSpot analysis [30]. There was no correlation between IL-2 production in response to spike peptide stimulation and time since infection, time since vaccination, age or when the plates were run (i.e. plate by plate variability). Donors who produced low IL-2 responses were as likely to produce high IFN-γ responses as those who produced high IL-2 responses, leaving us to conclude that this bimodal distribution is either coincidental to this cohort or due to a factor outside of our knowledge.

Peptide pools based on the predicted aa sequences from the original Wuhan strain of SARS-CoV-2 successfully identified people who were first infected with the Omicron variant of SARS-CoV-2 (Figs2  3). It is reassuring to note that peptides covering the original Wuhan strain of SARS-CoV-2 were also able to stimulate memory T cell responses, which were likely raised against infections with the Omicron (BA.1) strain. Likely, this is because the M and N aa sequences are fairly stable in SARS-CoV-2 with only 3/222 (1.3 %) and 8/416 (1.9 %) aa changes between published Wuhan and Omicron BA.1 strains (GenBank sequences MN908947.3 and OX315743.1, respectively).

Additionally, it is possible to use spike peptide pools in this assay to measure the efficacy of vaccination programmes, which Oliver *et al*. have already achieved using Luminex cytokine array data as a readout [34]. The presence of strong spike T cell responses in the absence of Nc and M responses may also suggest successful vaccination in uninfected individuals. Spike protein has mutated somewhat more than either M or N with 41/1273 (3.2 %) aa differences, and one might expect more genetic drift over time. If one wanted to use spike responses to track infection over time, results would right now be confounded by vaccination. Previous work suggests that the broad coverage of whole SARS-CoV-2 proteins means that individual point mutations in epitopes are unlikely to reduce T cell responses; however, a point mutation in one epitope can completely ablate T cell recognition of that epitope [54,55]. Therefore, it is hard to determine how many mutations the assay could tolerate without experimentally testing peptide pools for variants of concerns.

In this work and previously [30], some uninfected cohort samples still responded to SARS-CoV-2 peptides, a phenomenon likely caused by some peptides in the whole Nc and M peptide pools having sequences identical to or similar enough to circulating coronaviruses Nc and M proteins, leading to T cell cross-reactivity. Indeed, others have seen cross-reactive T cells in SARS-CoV-2-naïve, healthy controls [41,56,58]. We tested this hypothesis by restricting the peptide pool only to those peptides which have fewer than six aa overlaps with circulating coronaviruses NL63, 229E, OC43 or HKU1 (Table S1). These epitope-restricted pools only stimulated two members of the uninfected cohort samples and only when PBMCs were stimulated. No uninfected control cohort responded when the whole blood assay was used. This shows that the epitope-restricted peptide pool allows for greater selectivity and that cross-reactive T cells are a source of false-positive results. Somewhat paradoxically, the sensitivity of the assay increased when thawed PBMCs were stimulated with the epitope-restricted peptide pools compared to the whole peptide pools (Fig. 5e–h). The difference for IFN-γ production is only marginally improved (88% vs 90%) and so is likely to be within any margin of error. For IL-2, the responses were much lower (22% vs 50%) and would not be useful for diagnoses. As the threshold for a positive response is any IL-2 signal detected above background levels, the differences could be coincidental.

It cannot be determined with absolute certainty that none of the uninfected control cohort had been exposed to SARS-CoV-2. This is for many reasons including that the uninfected cohort was not constantly screened during the COVID-19 pandemic for infection, false-negative results can occur [17,18], some infections are asymptomatic [59,60] and not all infected donors maintain detectable anti-Nc antibodies [49]. Despite these limitations, the epitope-restricted peptide pool showed almost no stimulation of uninfected control samples, highlighting the likelihood that this cohort was indeed immunonaive to SARS-CoV-2.

Some infected cohort samples responded below the detectability threshold when stimulated with the epitope-restricted peptide pool compared with whole peptide sequence pools (Figs2  4). This result suggests that in some people, SARS-CoV-2 infection may predominantly re-stimulate existing cross-reactive T cells, which are specific for epitopes in coronavirus Nc and M proteins but generate *de novo* and thus memory T cell responses against new epitopes in others. Indeed, others have observed the induction of cross-reactive T cells after SARS-CoV-2 infection, and higher cross-reactive T cell numbers correlated with better disease outcomes [61]. The noted difference between different people could be caused by inherited HLA alleles, which would bias immune responses to specific epitopes [62]. The difference could also be due to the recent infections with circulating coronaviruses before COVID-19 [41,56, 63, 64].

Finally, this study showed that, in addition to fresh blood, this assay can also be used with PBMCs, which have been historically isolated from blood donations and then frozen in liquid nitrogen. Laboratories worldwide will have samples of frozen PBMCs from before the COVID-19 pandemic, allowing the analysis of immunity prior to SARS-CoV-2 exposure, which is increasingly difficult as majorities of most populations have been infected.

For FluoroSpot analysis, IL-2 secretion showed sensitivity and specificity of 83 and 75%, respectively, while IFN-γ showed 61 and 88% (Fig. 5a, b). This makes IL-2 more reliable than IFN-γ secretion for determining past SARS-CoV-2 infection by FluoroSpot analysis. However, IFN-γ was a better predictor than IL-2 when using ELISAs to measure cytokine production with sensitivity and specificity of 88/82% for IFN-γ against 22/90% for IL-2 (Fig. 5e, f). As the PBMCs were cultured at the same density and in the same media for both assays (TexMACS), the differences are likely due to the sensitivity of the assay formats used by proprietary FluoroSpot and ELISA kits, as well as the high IFN-γ background observed using FluoroSpot analysis in some people after COVID-19 [65].

Other groups have also compared different cytokines as readouts for past SARS-CoV-2 infections. Oliver *et al*. found that IL-2 was a better readout for past exposure than IFN-γ using whole blood stimulation. However, they used Bio-Rad’s Luminex cytokine array as a readout instead of ELISA [34]. Binayke *et al*. found similar results for IL-2 and IFN-γ as readout cytokines using ELISA, but marginally worse responses for TNF-α [46]. Scurr *et al*. found that IFN-γ has higher sensitivity but lower specificity than IL-2 [36]. Scurr *et al.*’s results are most similar to our own and also used the same proprietary ELISA kits to measure IL-2 and IFN-γ. This suggests that the differences between different T cell assays could be due to the differences in sensitivity between the antibodies used by different companies. Future studies will need to take these differences into account when developing clinical assays and carefully choose readout methods to maximize sensitivity and specificity.

If an approach similar to this was to be used as a diagnostic tool, we would propose testing patients with both the whole peptide and epitope-restricted peptide pools. Patients could be separated based on responses to both epitope-restricted peptide pools (strong positive), both whole peptide pools (weak positive) and anything less (negative). For some studies, high confidence in past SARS-CoV-2 exposure would be needed, so weak positive results could be excluded.

As the World Health Organization has confirmed 770 million COVID-19 cases as of November 2023, the utility of this assay needs to go beyond the current SARS-CoV-2 pandemic. Whole blood peptide stimulation could play a role in analysing the spread of future pandemic viruses. If another coronavirus pandemic were to occur, there is a possibility that it will have sequence similarity to SARS-CoV-2. In this case, the same approach can be used, stimulating blood with virus-specific epitopes, to avoid possible cross-reactivity with SARS-CoV-2. This work provides some confidence that this approach could be developed.

Taken together, this work highlights the efficacy of stimulating whole blood with peptides as a rapid and cheap method to measure SARS-CoV-2 T cell responses. This can be used to retrospectively determine SARS-CoV-2 exposure for epidemiological and other studies, either with fresh whole blood or using frozen PBMC samples. Epitope-restricted peptide pools can help increase confidence in diagnostic results, and the use of this method with frozen PBMCs widens the applicability of this method. For future pandemics, when measuring T cell responses, it is important to consider the readout cytokine one is using to optimize the efficacy of an assay.

## Supplementary material

10.1099/jgv.0.002055 Uncited Supplementary Material 1.

## Abbreviations

anti-Nc: anti-nucleocapsid
COVID-19: coronavirus disease 2019
M: membrane
Nc: nucleocapsid
PHA: phytohaemagglutinin
RT-qPCR: reverse transcription quantitative polymerase chain reaction
SARS-CoV-2: severe acute respiratory syndrome coronavirus 2
SFU: spot forming unit
