# Supplementary material for: Comparison of different T cell assays for the retrospective determination of SARS-CoV-2 infection
Source: J Gen Virol. 2024 Dec 20;105(12):002055. doi: 10.1099/jgv.0.002055 (PMC12453407; doi:10.1099/jgv.0.002055)
Supplement: Uncited Supplementary Material 1. [file jgv-105-02055-s001.pdf]

Supplementary Table S1

Nucleocapsid peptides

MSDNGPQNQRNAPRI	SPRWYFYYLGTGPEA	RMAGNGGDAALALLL	AFFGMSRIGMEVTPS
GPQNQRNAPRITFGG	YFYYLGTGPEAGLPY	NGGDAALALLLLDRL	MSRIGMEVTPSGTWL
QRNAPRITFGGPSDS	LGTGPEAGLPYGANK	AALALLLLDRLNQLE	GMEVTPSGTWLTYTG
PRITFGGPSDSTGSN	PEAGLPYGANKDGII	LLLLDRLNQLESKMS	TPSGTWLTYTGAIKL
FGGPSDSTGSNQNGE	LPYGANKDGIIWVAT	DRLNQLESKMSGKGQ	TWLTYTGAIKLDDKD
SDSTGSNQNGERSGA	ANKDGIIWVATEGAL	QLESKMSGKGQQQQG	YTGAIKLDDKDPNFK
GSNQNGERSGARSKQ	GIIWVATEGALNTPK	KMSGKGQQQQGQTVT	IKLDDKDPNFKDQVI
NGERSGARSKQRRPQ	VATEGALNTPKDHIG	KGQQQQGQTVTKKSA	DKDPNFKDQVILLNK
SGARSKQRRPQGLPN	GALNTPKDHIGTRNP	QQGQTVTKKSAAEAS	NFKDQVILLNKHIDA
SKQRRPQGLPNNTAS	TPKDHIGTRNPANNA	TVTKKSAAEASKKPR	QVILLNKHIDAYKTF
RPQGLPNNTASWFTA	HIGTRNPANNAAIVL	KSAAEASKKPRQKRT	LNKHIDAYKTFPPTE
LPNNTASWFTALTQH	RNPANNAAIVLQLPQ	EASKKPRQKRTATKA	IDAYKTFPPTEPKKD
TASWFTALTQHGKED	NNAAIVLQLPQGTTL	KPRQKRTATKAYNVT	KTFPPTEPKKDKKKK
FTALTQHGKEDLKFP	IVLQLPQGTTLPKGF	KRTATKAYNVTQAFG	PTEPKKDKKKKADET
TQHGKEDLKFPRGQG	LPQGTTLPKGFYAEG	TKAYNVTQAFGRRGP	KKDKKKKADETQALP
KEDLKFPRGQGVPIN	TTLPKGFYAEGSRGG	NVTQAFGRRGPEQTQ	KKKADETQALPQRQK
KFPRGQGVPINTNSS	KGFYAEGSRGGSQAS	AFGRRGPEQTQGNFG	DETQALPQRQKKQQT
GQGVPINTNSSPDDQ	AEGSRGGSQASSRSS	RGPEQTQGNFGDQEL	ALPQRQKKQQTVTLL
PINTNSSPDDQIGYY	RGGSQASSRSSSRSR	QTQGNFGDQELIRQG	RQKKQQTVTLLPAAD
NSSPDDQIGYYRRAT	QASSRSSSRSRNSSR	NFGDQELIRQGTDYK	QQTVTLLPAADLDDF
DDQIGYYRRATRRIR	RSSSRSRNSSRNSTP	QELIRQGTDYKHWPQ	TLLPAADLDDFSKQL
GYYRRATRRIRGGDG	RSRNSSRNSTPGSSR	RQGTDYKHWPQIAQF	AADLDDFSKQLQQSM
RATRRIRGGDGKMKD	SSRNSTPGSSRGTSP	DYKHWPQIAQFAPSA	DDFSKQLQQSMSSAD
RIRGGDGKMKDLSPR	STPGSSRGTSPARMA	WPQIAQFAPSASAFF	KQLQQSMSSADSTQA
GDGKMKDLSPRWYFY	SSRGTSPARMAGNGG	AQFAPSASAFFGMSR	
MKDLSPRWYFYYLGT	TSPARMAGNGGDAAL	PSASAFFGMSRIGME	

Membrane protein

MADSNGTITVEELKK	FLWLLWPVTLACFVL	RTRSMWSFNPETNIL	GRCDIKDLPKEITVAT
NGTITVEELKKLLEQ	LWPVTLACFVLAAVY	MWSFNPETNILLNVP	IKDLPKEITVATSRTL
TVEELKKLLEQWNLV	TLACFVLAAVYRINW	NPETNILLNVPLHGT	PKEITVATSRTLSYYK
LKKLLEQWNLVIGFL	FVLAAVYRINWITGG	NILLNVPLHGTILTR	VATSRTLSYYKLGAS
LEQWNLVIGFLFLTW	AVYRINWITGGIAIA	NVPLHGTILTRPLLE	RTLSYYKLGASQRVA
NLVIGFLFLTWICLL	INWITGGIAIAMACL	HGTILTRPLLESELV	SYYKLGASQRVAGDSG
GFLFLTWICLLQFAY	TGGIAIAMACLVGLM	LTRPLLESELVIGAV	LGASQRVAGDSGFAAY
LTWICLLQFAYANRN	AIAMACLVGLMWLSY	LLESELVIGAVILRG	QRVAGDSGFAAYSRYR
CLLQFAYANRNRFLY	ACLVGLMWLSYFIAS	ELVIGAVILRGHLRI	DSGFAAYSRYRIGNY
FAYANRNRFLYIIKL	GLMWLSYFIASFRLF	GAVILRGHLRIAGHH	FAAYSRYRIGNYKLNT
NRNRFLYIIKLIFLW	LSYFIASFRLFARTR	LRGHLRIAGHHLGRC	SRYRIGNYKLNTDHSS
FLYIIKLIFLWLLWP	IASFRLFARTRSMWS	LRIAGHHLGRCDIKD	IGNYKLNTDHSSSSDN
IKLIFLWLLWPVTLA	RLFARTRSMWSFNPE	GHHLGRCDIKDLPKE	KLNTDHSSSSDNIALL
DHSSSSDNIALLVQ			

Supplementary Figures

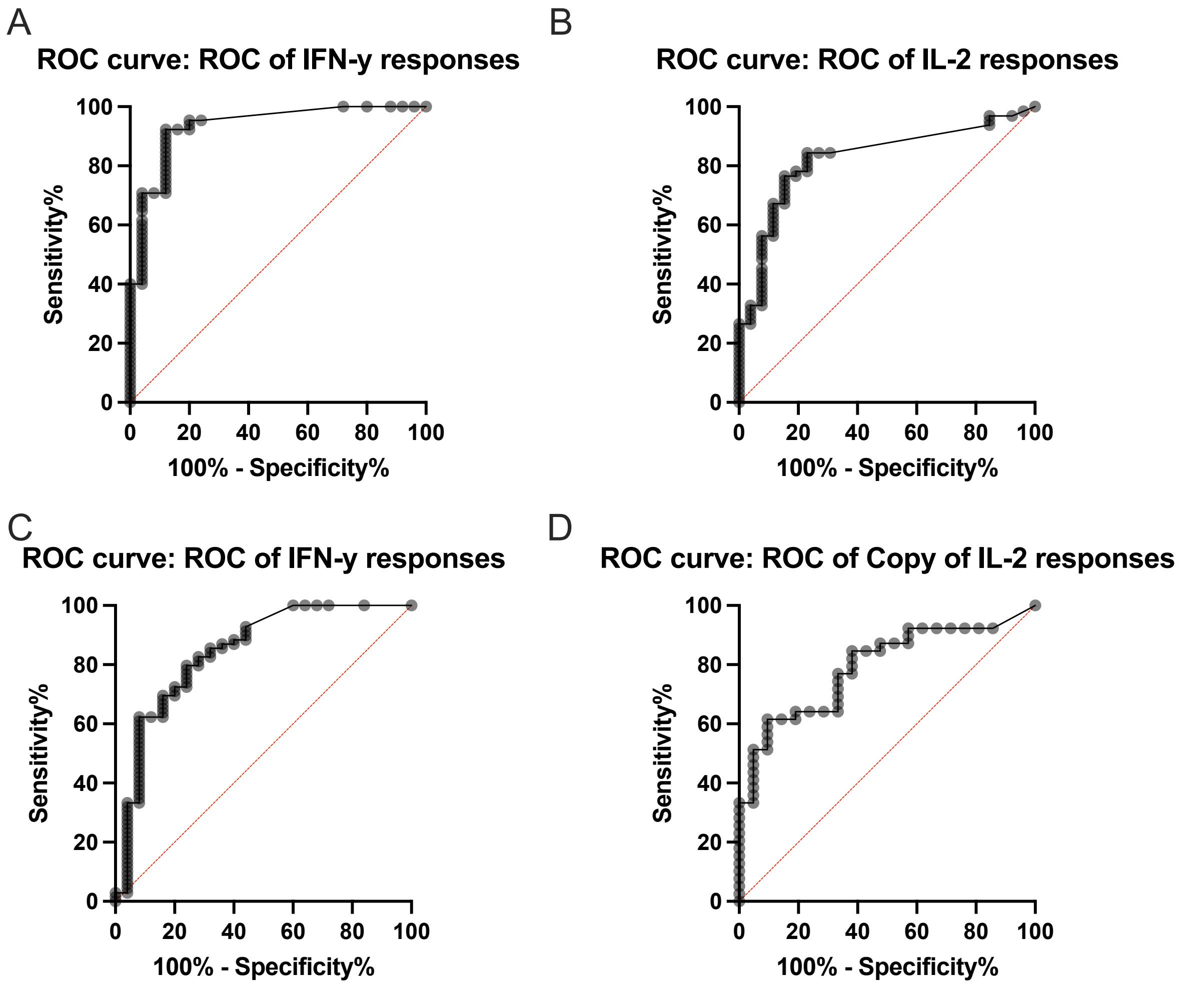

Figure S1: ROC curves for Figure 2A&B showing IL-2 and IFN-y responses to Nucleocapsid peptides (A&B) and membrane peptides (C&D), which were used to calculate the positive/negative thresholds shown in the figure as dotted lines.

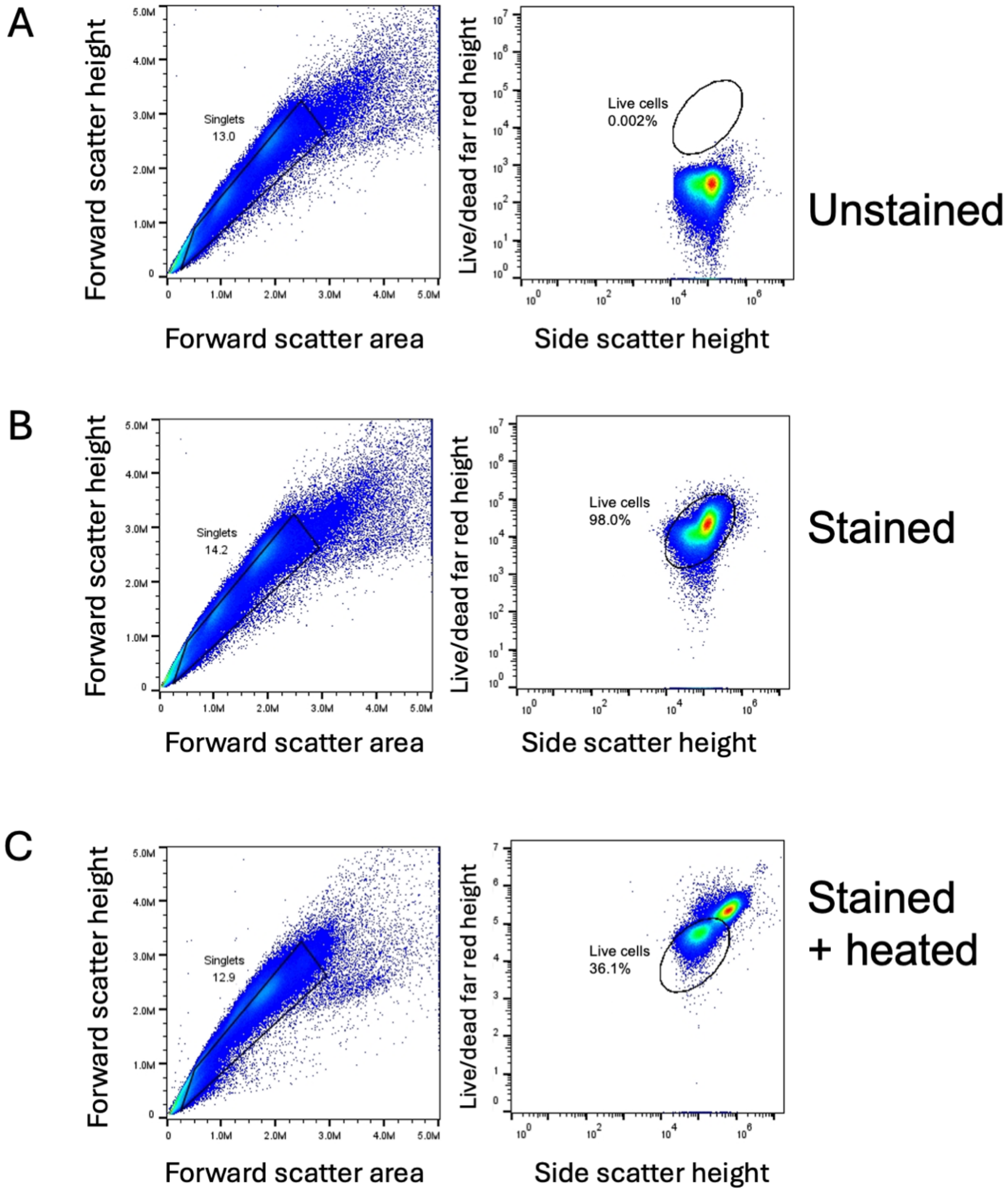

Figure S2: Representation of experimental procedure. Representative dot plots from 1 donor are shown illustrating the gating strategy for calculating the number of PBMCs loaded per well. In short, PBMCs were stained for survival using Live/Dead Far red dye. Singlet cells were selected based on forward scatter height and area (left) and cells within this gate were then analysed for Live/Dead dye uptake, and those within the indicated gate were counted as live PBMCs. To demonstrate the efficacy of the staining approach, unstained control cells (A), live cells (B) and cells killed by heating to 80 °C for 20 mins (C) were run.

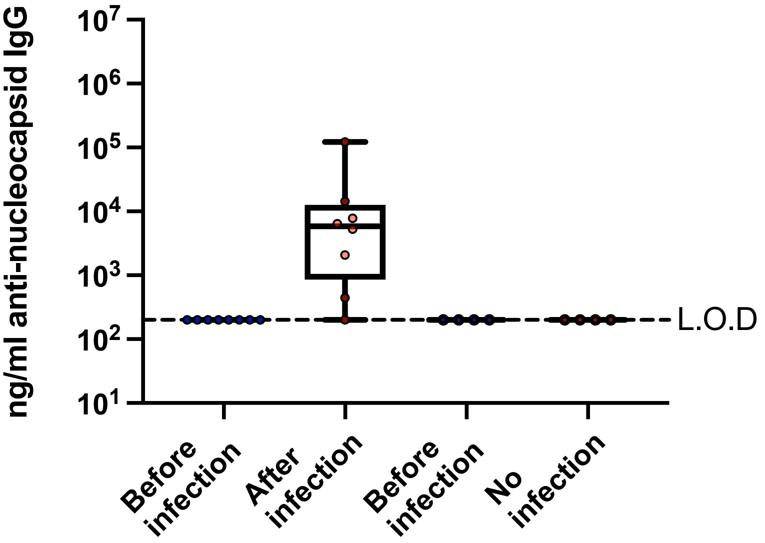

Figure S3: Most blood donors who were initially uninfected produced anti-Nc antibodies after infection. To determine if Omicron infection induced anti-nucleocapsid antibodies, antibody levels were measured by ELISA from diluted plasma. Samples which were below the detectability threshold were set to 200 ng/ul.
